# Adenomyoma of the ampulla of Vater mimicking malignancy: A case report and literature review

**DOI:** 10.1097/MD.0000000000034080

**Published:** 2023-06-16

**Authors:** Hyung Jun Kwon, Sang Geol Kim, Jinyoung Park

**Affiliations:** a Department of Surgery, School of Medicine, Kyungpook National University, Kyungpook National University Hospital, Daegu, South Korea.

**Keywords:** adenomyoma, ampulla of Vater, case report, pancreatoduodenectomy

## Abstract

Adenomyoma is a rare reactive, hamartomatous benign tumor-like lesion. Although adenomyoma can occur anywhere in the gastrointestinal tract, including the gallbladder, stomach, duodenum, and jejunum, it is very rarely observed in the extrahepatic bile duct and ampulla of Vater (AOV). The preoperative accurate diagnosis of adenomyoma of the Vaterian system, including the AOV and common bile duct, is significant to appropriate patient management. However, discriminating between benign and malignancy is highly challenging. Patients are frequently mistaken as having periampullary malignancy, thereby leading to unnecessary extensive surgical resection with a high risk of complications.

**Patient concerns:**

A 47-year-old woman visited a local hospital owing to epigastric and right upper-quadrant abdominal pain for 2 days.

**Diagnoses:**

Abdominal ultrasonography performed in the local hospital revealed suspicious of a distal common bile duct malignancy. She was transferred to our hospital for further evaluation and management.

**Interventions:**

After consulting with the patient, a multidisciplinary team, including a gastroenterologist, finally decided to perform surgery under the impression of an ampullary malignancy, and pylorus-preserving pancreatoduodenectomy was performed without any complications. She was histopathologically diagnosed with an adenomyoma of the AOV.

**Outcomes:**

At the 5-year follow-up, she was well and did not develop further symptoms or complications.

**Lessons:**

Although adenomyoma is very rare, it should be included in the differential diagnosis of mass-like lesions of the AOV to avoid unnecessary surgeries.

## 1. Introduction

Adenomyoma is a rare reactive, hamartomatous benign tumor-like lesion.^[1,2]^ Although it can occur anywhere in the gastrointestinal tract, including the gallbladder, stomach, duodenum, and jejunum, it is very rarely observed in the extrahepatic bile duct and ampulla of Vater (AOV).^[3–5]^ The preoperative accurate diagnosis of adenomyoma of the Vaterian system, including the AOV and common bile duct (CBD), is significant to appropriate patient management. However, discriminating between benign and malignancy is highly challenging. Patients are frequently mistaken as having periampullary malignancy, thereby leading to unnecessarily extensive surgical resection with a high risk of complications. We report a 47-year-old woman with adenomyoma of the AOV who was treated with pylorus-preserving pancreatoduodenectomy without any complications.

## 2. Case report

**Figure 1.**
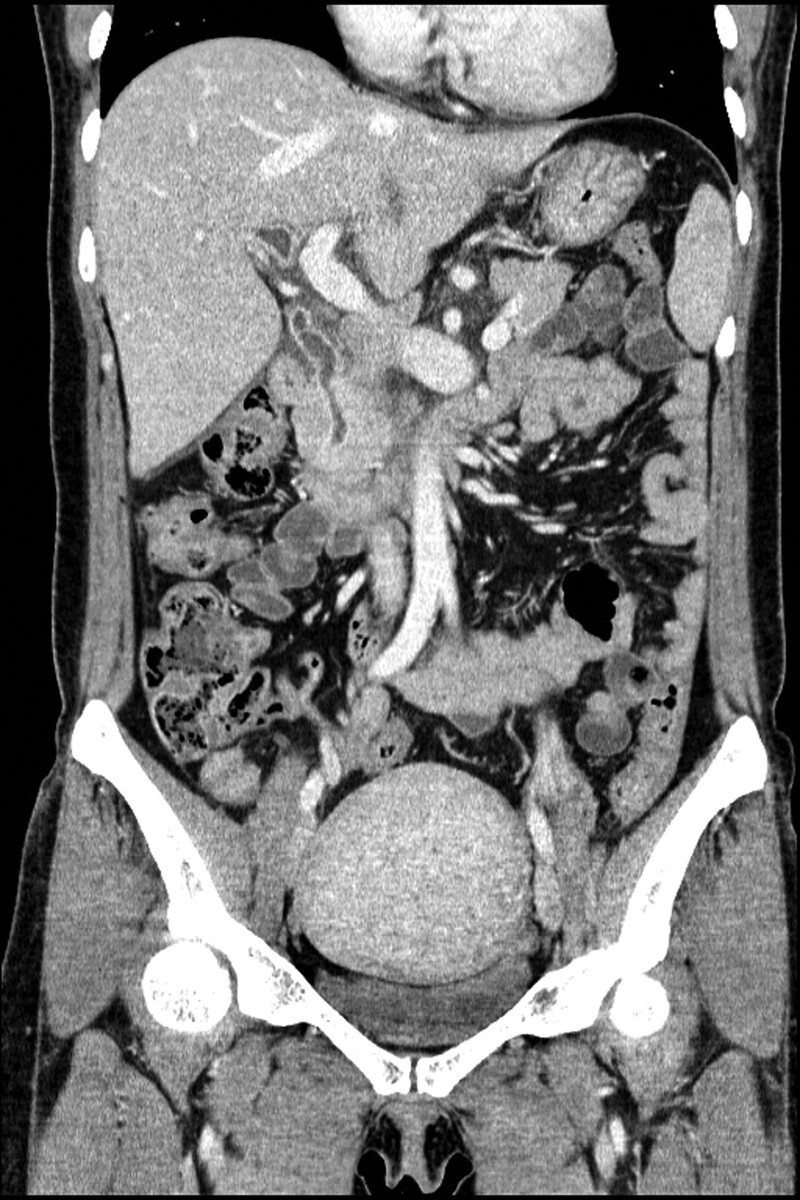
Coronal view of abdominal computed tomography shows mild extrahepatic bile duct dilatation with diffuse wall thickening and enhancement.

**Figure 2.**
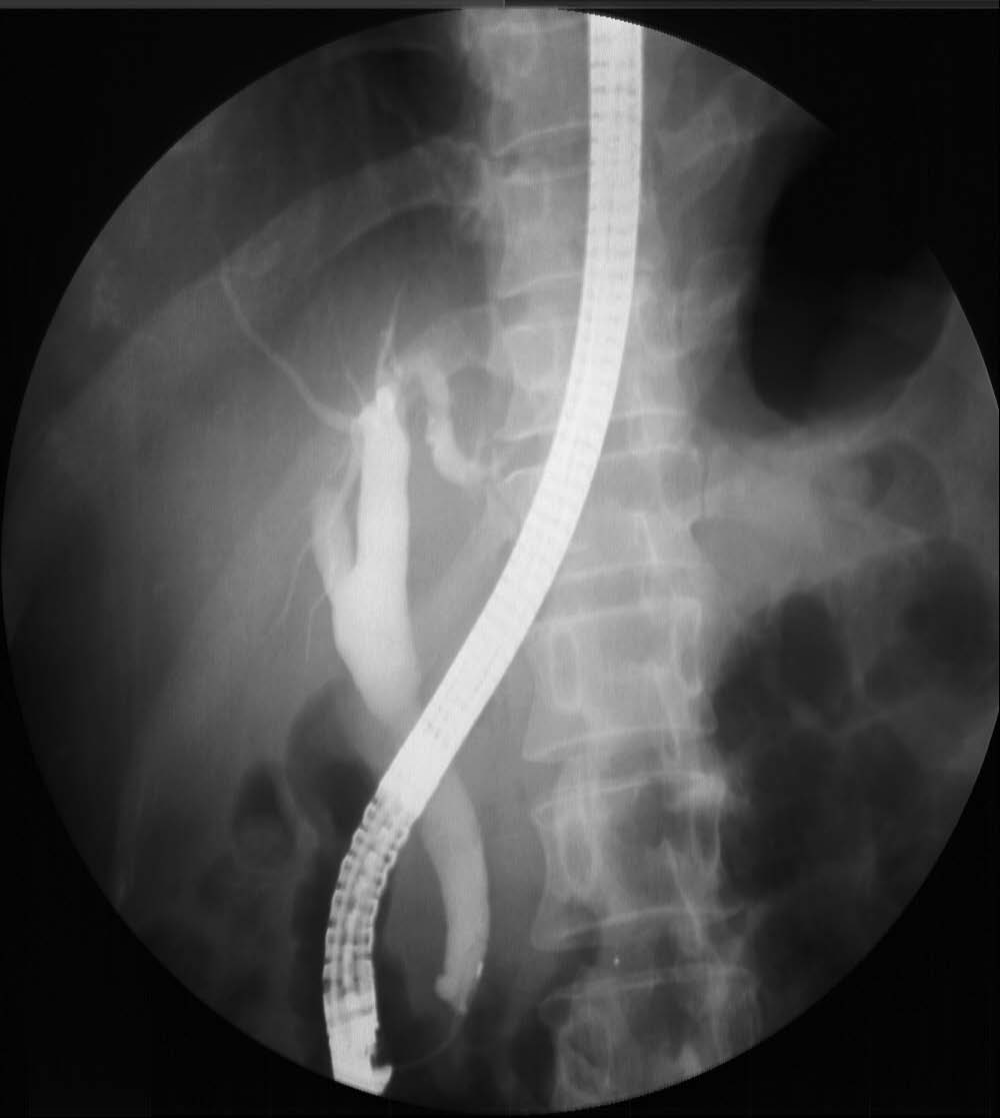
Endoscopic retrograde cholangiopancreatography reveals proximal biliary duct dilatation and smooth tapering of the distal CBD without filling defects and luminal obstruction at the distal CBD. CBD = common bile duct.

**Figure 3.**
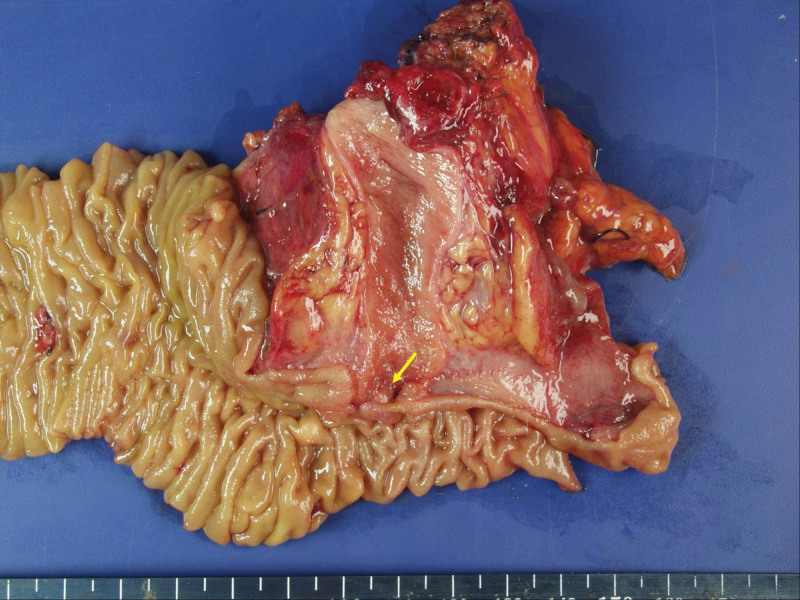
The resected specimen has a 10 × 5-mm-sized, submucosal polypoid lesion (yellow arrow) in the AOV. AOV = ampulla of Vater.

A 47-year-old woman visited a local hospital owing to epigastric and right upper quadrant (RUQ) abdominal pain for 2 days. Abdominal ultrasonography performed in the local hospital revealed intrahepatic and extrahepatic bile duct dilatation suspicious of a distal CBD malignancy. She was transferred to our hospital for further evaluation and management. She underwent cholecystectomy due to gallstone 20 years ago. Her personal and family medical histories were unremarkable. She complained of epigastric and RUQ abdominal pain. The following were her vital signs at the time of arrival: blood pressure, 117/71 mm Hg; heart rate, 87 beats/min; respiratory rate, 16 cycles/min; and body temperature, 37.4℃. On physical examination, mild icteric sclera and epigastric and RUQ abdominal tenderness were noted. Initial laboratory findings showed normal values for her white blood cell count, hemoglobin, platelet, and erythrocyte sedimentation rate, except an elevated C-reactive protein level of 2.23 mg/dL. The following were her serum liver enzyme levels: aspartate transaminase, 95 U/L; alanine transaminase, 165 U/L; total bilirubin, 5.83 mg/dL; conjugated bilirubin, 4.7 mg/dL; alkaline phosphatase, 317 U/L; and γ-glutamyltransferase, 650 U/L. Serum amylase and lipase levels were 24 and 181 U/L, respectively. The carcinoembryonic antigen level was 1.3 ng/mL, and the CA 19-9 level was increased to 97.6 U/mL. Abdominal ultrasonography revealed mild intrahepatic and extrahepatic bile duct dilatation with diffuse wall thickening and smooth distal CBD tapering. Furthermore, abdominal computed tomography revealed mild extrahepatic bile duct dilatation with diffuse wall thickening and enhancement (Fig. 1). Endoscopic retrograde cholangiopancreatography (ERCP) demonstrated proximal biliary duct dilatation and smooth distal CBD tapering without filling defects and luminal obstruction at the distal CBD (Fig. 2). With an impression of biliary sludge, endoscopic nasobiliary drainage (ENBD) was placed. Laboratory test performed on the day following ENBD showed decreased total bilirubin and conjugated bilirubin levels of 1.43 and 1.14 mg/dL, respectively. Moreover, aspartate transaminase and alanine transaminase levels decreased to 43 and 94 U/L, respectively. Follow-up cholangiogram through the ENBD performed on the fourth day following ENBD still revealed extrahepatic biliary duct dilatation. Endoscopy with sphincterotomy revealed an intraampullary polypoid lesion, and biopsy was subsequently performed. Histopathologic examination from endoscopic biopsy revealed no atypical epithelial cells with chronic inflammation of the ampullary region. Although the endoscopic biopsy result did not show any evidence of malignancy, the possibility of malignancy could not be completely excluded owing to the increased CA 19-9 level and clinical findings. Therefore, after consulting with the patient, a multidisciplinary team, including a gastroenterologist, finally decided to perform surgery under the impression of an ampullary malignancy, and pylorus-preserving pancreatoduodenectomy was performed without any complications. The resected specimen had a 10 × 5-mm-sized, submucosal polypoid lesion in the AOV (Fig. 3). The postoperative course was uneventful. Microscopically, the lesion consisted of a lobular arrangement of benign glands and proliferated smooth muscle fibers. She was histopathologically diagnosed with an adenomyoma of the AOV. At the 5-year follow-up, she was well and did not develop further symptoms or complications.

## 3. Discussion

Adenomyoma is a rare reactive, hamartomatous benign tumor-like lesion.^[1,2]^ Although it can occur anywhere in the gastrointestinal tract, including the gallbladder, stomach, duodenum, and jejunum, it is very rarely observed in the extrahepatic bile duct and AOV.^[3–5]^ Hammarström et al reported that of 3131 ERCP cases, 4 patients were diagnosed with ampullary adenomyoma, and the incidence of adenomyoma of the AOV was 0.13%.^[1]^ Women have a 3-fold higher prevalence of adenomyoma than men, with a median patient age of 67 years.^[2]^

The exact mechanism of the histogenesis of adenomyoma in the Vaterian system remains unclear. The most widely accepted hypothesis is that these lesions may represent a form of an incomplete heterotopic pancreas. Another hypothesis is that the dysfunction of the muscle fibers of the AOV due to chronic papillitis may lead to muscular hyperplasia or adenomyomatous hyperplasia.^[2]^

Adenomyoma is histologically characterized by a dense proliferation of ducts and ductules with smooth muscle bundles and collagen fibers. Multiple lobules of the glands are mainly located in the muscular layers of the Vaterian system, thereby resulting in the hypertrophy of the sphincter of Oddi.^[2]^ Furthermore, it has immunohistochemical characteristics of cytokeratin 7 expression without cytokeratin 20 expression.^[2]^

Adenomyoma of the AOV grows gradually and asymptomatically for a long period. Depending on the size of the lesion, the symptoms of adenomyoma of the AOV include RUQ pain, dysphagia, obstructive jaundice with or without cholangitis, and acute pancreatitis with clinical similarities to periampullary tumors.^[5–7]^

The preoperative accurate diagnosis of adenomyoma of the Vaterian system, including the AOV and CBD, is significant to appropriate patient management. However, discriminating between benign and malignancy is highly challenging. Patients are frequently mistaken as having periampullary malignancy, thereby leading to unnecessarily extensive surgical resection with a high risk of complications, including pancreatic fistula and delayed gastric emptying. Preoperative imaging modalities, such as computed tomography and magnetic resonance imaging, could be utilized to reveal CBD obstruction or well-demarcated mass lesions in the ampullary area. Additionally, ERCP is typically performed to allow direct inspection of the periampullary region. Furthermore, histopathological examination via endoscopic biopsies may be helpful in superficial lesions although could not definitely exclude underlying ampullary malignant lesions located deeper with a low diagnostic yield.^[3]^ Menzel et al^[8]^ reported that endoscopic forceps biopsies did not allow for reliable preoperative diagnosis of tumors of the AOV. Moreover, they recommended that in case of enlarged or suspicious papillae, endoscopic biopsies from deep and superficial layers immediately following sphincterotomy should be obtained to improve the diagnostic accuracy of endoscopic biopsies.

Deciding which treatment option to select for ampullary lesions before surgery is highly challenging since radiologic imaging and endoscopic investigation may not be able to discriminate between benign and malignancy in the ampullary lesion. It has been reported that endoscopic ampullectomy or transduodenal papillectomy could be sufficient for the treatment of adenomyoma of the AOV.^[6,7,9]^ Therefore, accurate preoperative and intraoperative diagnoses, including histological evaluation, are mandatory for a definite diagnosis and prevent unnecessary extensive surgeries as in this case.^[10]^

## 4. Conclusion

Although adenomyoma is very rare, it should be included in the differential diagnosis of mass-like lesions of the AOV to avoid unnecessary surgeries.

## Author contributions

**Conceptualization:** Hyung Jun Kwon, Sang Geol Kim, Jinyoung Park.

**Formal analysis:** Hyung Jun Kwon, Sang Geol Kim, Jinyoung Park.

**Writing – original draft:** Hyung Jun Kwon, Jinyoung Park.

**Writing – review & editing:** Jinyoung Park.
